# Supplementary material for: Distinct signals in medial and lateral VTA dopamine neurons modulate fear extinction at different times
Source: eLife. 2020 Jun 10;9:e54936. doi: 10.7554/eLife.54936 (PMC7363446; doi:10.7554/eLife.54936)
Supplement: Transparent reporting form [file elife-54936-transrepform.pdf]

eLIFE
1st Floor
24 Hills Road
Cambridge CB2 1JP, UK
P 01223 855340
W elifesciences.org
T @elife

eLife's transparent reporting form

We encourage authors to provide detailed information *within their submission* to facilitate the interpretation and replication of experiments. Authors can upload supporting documentation to indicate the use of appropriate reporting guidelines for health-related research (see EQUATOR Network), life science research (see the BioSharing Information Resource), or the ARRIVE guidelines for reporting work involving animal research. Where applicable, authors should refer to any relevant reporting standards documents in this form.

If you have any questions, please consult our Journal Policies and/or contact us: editorial@elifesciences.org.

Sample-size estimation

- You should state whether an appropriate sample size was computed when the study was being designed
- You should state the statistical method of sample size computation and any required assumptions
- If no explicit power analysis was used, you should describe how you decided what sample (replicate) size (number) to use

Please outline where this information can be found within the submission (e.g., sections or figure legends), or explain why this information doesn't apply to your submission:

No explicit power analysis was used to determine sample size for fiber photometry and optogenetics experiments. We decided the sample size (N is approximately 10 for each group) based on what is typically seen in the literature.
This information can be found in the fiber photometry and optogenetics Methods section.

Replicates

- You should report how often each experiment was performed
- You should include a definition of biological versus technical replication
- The data obtained should be provided and sufficient information should be provided to indicate the number of independent biological and/or technical replicates
- If you encountered any outliers, you should describe how these were handled
- Criteria for exclusion/inclusion of data should be clearly stated
- High-throughput sequence data should be uploaded before submission, with a private link for reviewers provided (these are available from both GEO and ArrayExpress)

Please outline where this information can be found within the submission (e.g., sections or figure legends), or explain why this information doesn't apply to your submission:

eLife Sciences Publications, Ltd is a limited liability non-profit non-stock corporation incorporated in the State of Delaware, USA, with company number 5030732, and is registered in the UK with company number FC030576 and branch number BR015634 at the address 1st Floor, 24 Hills Road, Cambridge CB2 1JP | August 2014

No outliers were discarded. The sample size of approximately N of 10 per group are biological replicates, where biological replicate means an individual mouse. For two of the three optogenetic cohorts, two different rounds of mice were run on separate occasions and data was combined for the full cohort. For the third cohort, all mice were run at once. For fiber photometry cohorts, 1-4 mice were run on each occasion and all data was aggregated to create the cohorts for the paper.
This information can be found in the fiber photometry and optogenetics Methods section.

Statistical reporting

- Statistical analysis methods should be described and justified
- Raw data should be presented in figures whenever informative to do so (typically when N per group is less than 10)
- For each experiment, you should identify the statistical tests used, exact values of N, definitions of center, methods of multiple test correction, and dispersion and precision measures (e.g., mean, median, SD, SEM, confidence intervals; and, for the major substantive results, a measure of effect size (e.g., Pearson's r, Cohen's d)
- Report exact p-values wherever possible alongside the summary statistics and 95% confidence intervals. These should be reported for all key questions and not only when the p-value is less than 0.05.

Please outline where this information can be found within the submission (e.g., sections or figure legends), or explain why this information doesn't apply to your submission:

For each experiment, we identify the statistical test used, exact N values, and methods of multiple test correction if relevant. We report all p-values (even when not significant) alongside standard error of the mean, or standard deviation.
This information can be found in each figure, its legend, or the main text that references that figure. Details of statistical analyses used are further described in the Methods section.

(For large datasets, or papers with a very large number of statistical tests, you may upload a single table file with tests, Ns, etc., with reference to sections in the manuscript.)

Group allocation

- Indicate how samples were allocated into experimental groups (in the case of clinical studies, please specify allocation to treatment method); if randomization was used, please also state if restricted randomization was applied
- Indicate if masking was used during group allocation, data collection and/or data analysis

Please outline where this information can be found within the submission (e.g., sections or figure legends), or explain why this information doesn't apply to your submission:

For optogenetic experiments, littermates within a cage were randomly allocated to either NpHR or YFP group, with as equal distribution as possible. (ie. In a cage with 4 mice: 2 mice were in NpHR group, 2 mice in YFP group. In a cage with 5 mice, 2 mice were in NpHR group and 3 mice in YFP group or vice versa.) Masking was not used during allocation or data collection; this ensured that NpHR and YFP mice were interleaved during the experimental sessions and counterbalanced in different behavioral chambers. Masking was a central part of the behavioral analysis: the CNN automatically scores freezing the same regardless of the mouse's experimental group.
This information can be found in the Methods section.

Additional data files ("source data")

- We encourage you to upload relevant additional data files, such as numerical data that are represented as a graph in a figure, or as a summary table
- Where provided, these should be in the most useful format, and they can be uploaded as "Source data" files linked to a main figure or table
- Include model definition files including the full list of parameters used
- Include code used for data analysis (e.g., R, MatLab)
- Avoid stating that data files are "available upon request"

Please indicate the figures or tables for which source data files have been provided:

With final submission (if accepted), we will provide the source data and code for figures. We will also share the Github repository with the CNN pipeline code.
